# The Burden of Chikungunya in India: An Overview of Clinical, Epidemiological, and Public Health Challenges

**DOI:** 10.7759/cureus.108409

**Published:** 2026-05-07

**Authors:** Rohit R Patil, Satyajeet Pawar, Satish Patil

**Affiliations:** 1 Department of Molecular Biology and Genetics, Krishna Institute of Medical Sciences, Krishna Vishwa Vidyapeeth (Deemed to Be University), Karad, IND; 2 Department of Microbiology, Krishna Institute of Medical Sciences, Krishna Vishwa Vidyapeeth (Deemed to Be University), Karad, IND

**Keywords:** chikungunya virus, climate change and arboviruses, epidemiology of chikungunya in india, mosquito borne diseases, public health challenges, socio-economic burden

## Abstract

Chikungunya virus (CHIKV) is an arbovirus of significant public health concern, primarily transmitted by *Aedes* mosquitoes. A review of literature from sources such as PubMed, SCOPUS, and Web of Science indicates that India has faced several outbreaks in recent decades, characterised by high morbidity, an economic burden, and long-term complications. The virus’s re-emergence after periods of low transmission exposes weaknesses in surveillance, vector control, and community awareness. The increasing burden of chikungunya can be attributed to factors such as urbanisation, population movement, climate change, and a weak healthcare infrastructure. Diagnosing the disease is further complicated by symptoms that overlap with those of dengue and Zika, leading to frequent underreporting and misidentification. While outbreaks, especially in densely populated urban areas, rarely result in fatalities, the debilitating joint pain commonly associated with the virus can persist for months, significantly impacting quality of life and productivity. Epidemiological studies indicate cyclical patterns of transmission, typically aligning with the monsoon seasons and ineffective vector management. Various challenges, including gaps in diagnostics, limited laboratory capacity, and insufficient reporting systems, hinder timely responses to outbreaks.

This review aims to provide a comprehensive summary of the epidemiology, clinical manifestations, immunological characteristics, challenges in surveillance, and public health impacts of chikungunya in India, with a focus on recent trends and future research needs. Challenges include limited vaccine availability and implementation, a lack of community awareness, insufficient funding, inconsistent vector control efforts, underutilisation of molecular diagnostics such as reverse transcription-polymerase chain reaction (RT-PCR), and weak surveillance systems that lead to underreporting and delayed outbreak responses. To mitigate the long-term burden of chikungunya, it is essential to strengthen surveillance, expand diagnostic facilities, invest in research on the virus's origin and vaccine development, and implement community-based vector control measures.

## Introduction and background

Chikungunya is a re-emerging viral disease transmitted primarily by the *Aedes aegypti *and *Aedes albopictus* mosquitoes. It is caused by the chikungunya virus (CHIKV) and is characterised by symptoms such as acute fever, severe joint pain (arthralgia), rash, headache, and fatigue. Although chikungunya rarely leads to death, it can result in significant morbidity due to persistent musculoskeletal pain. Many patients continue to experience chronic joint pain even after recovery, which can lead to a reduced quality of life and functional disability [1]. The World Health Organisation defines suspected chikungunya cases as individuals presenting with acute fever and severe joint pain, with laboratory confirmation generally achieved through reverse transcription-polymerase chain reaction (RT-PCR) or serological testing [2].

In recent decades, chikungunya has reappeared as a significant global public health concern, with outbreaks reported across Asia, Africa, Europe, and the Americas. The geographical spread of CHIKV has been associated with climate variability, increased urbanisation, global travel, and the adaptation of mosquito vectors to new ecological environments [3]. In India, chikungunya outbreaks frequently follow a seasonal pattern, with increased transmission during the monsoon and post-monsoon periods, contributing to recurrent disease burden.

A major challenge in chikungunya-endemic regions is the clinical similarity between chikungunya and dengue and Zika infections, leading to frequent misdiagnosis. Limitations in molecular diagnostics, inadequate lab infrastructure, and weak surveillance systems delay outbreak recognition and result in underreporting. The lack of specific antiviral treatments and limited vaccine availability complicate prevention and control efforts [2,3].

This narrative review provides an overview of chikungunya in India, focusing on its epidemiology, clinical manifestations, virology, transmission dynamics, and major public health challenges. Additionally, it highlights the impact of climate change, urbanisation, and gaps in surveillance systems on the ongoing re-emergence of chikungunya. The review also emphasises future research priorities for effective disease prevention and control.

## Review

Search strategy

The literature search for this narrative review was conducted from November 2025 to March 2026 using the databases PubMed, Scopus, Web of Science, and Google Scholar. We included articles published between January 2005 and March 2026. The search strategy utilised the following keywords and Boolean combinations: (“Chikungunya” OR “CHIKV”) AND (“India” OR “epidemiology” OR “outbreak” OR “clinical manifestations” OR “arthralgia” OR “diagnosis” OR “RT-PCR” OR “ELISA” OR “Aedes aegypti” OR “Aedes albopictus” OR “vector control” OR “vaccine”). Additionally, we identified relevant publications through manual screening of the reference lists of selected articles. We also reviewed surveillance reports and official documents from the WHO, the National Vector Borne Disease Control Programme (NVBDCP), the Indian Council of Medical Research (ICMR), and the Ministry of Health and Family Welfare, Government of India. Only English-language publications were included in this review. Studies were eligible for inclusion if they addressed chikungunya epidemiology, virology, transmission, clinical outcomes, diagnostics, vector control, or public health impact, specifically focusing on India. We excluded case reports and studies involving single patients unless they provided significant diagnostic or clinical insights. Initially, we screened approximately 140 titles and abstracts, followed by a full-text evaluation of relevant studies. A total of 37 articles and reports were included in the final narrative synthesis.

Global epidemiology

**Figure 1:**
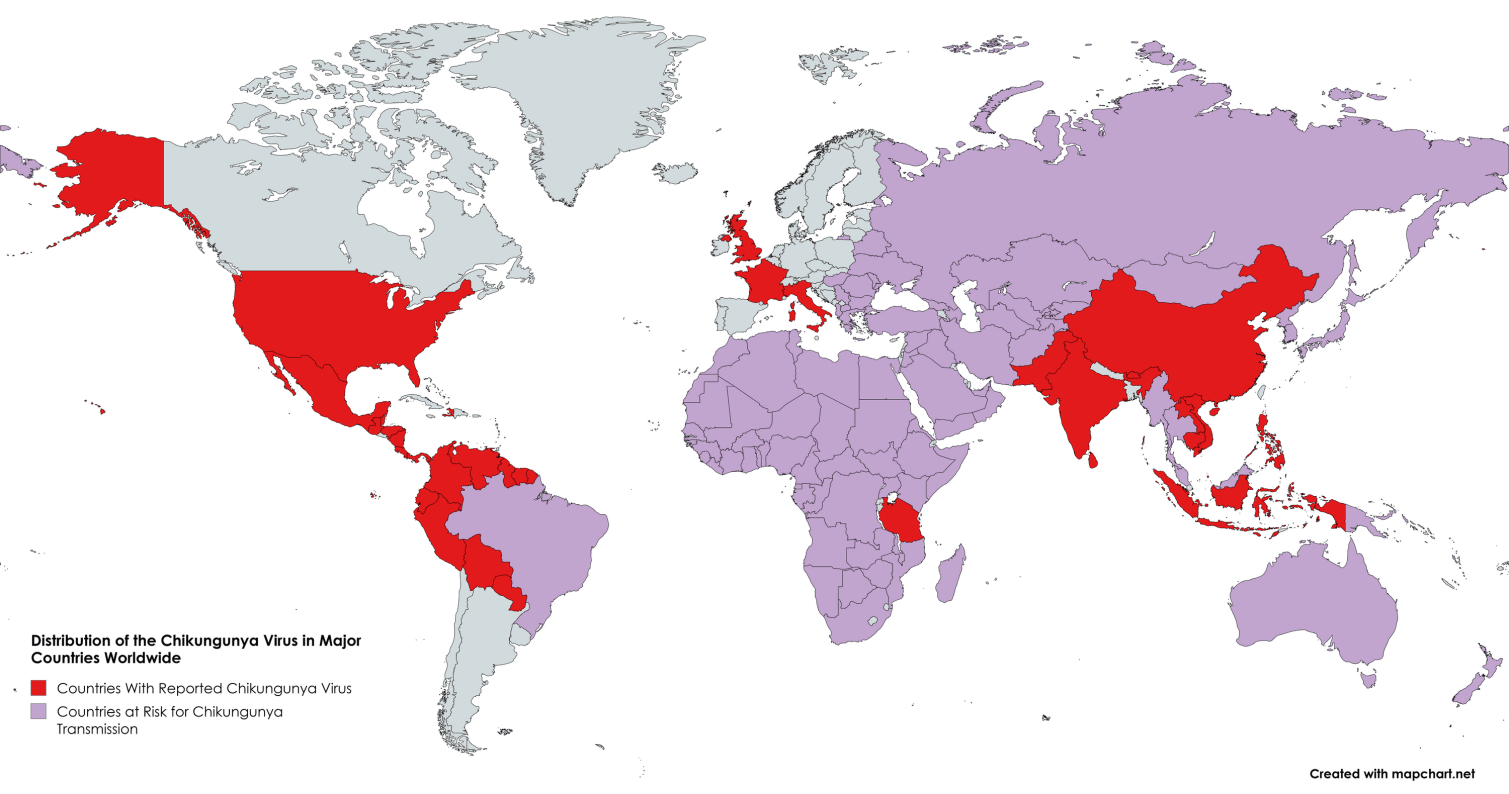
Distribution of the chikungunya virus in major countries worldwide. Source: Figure created by the authors using MapChart.net.

Chikungunya has been reported in more than 100 countries across Asia, Africa, Europe, and the Americas, indicating widespread global transmission as shown in Figure 1 [4-6]. The virus was first identified in Tanzania in 1952 and has since caused numerous outbreaks [7]. Between 2013 and 2014, a significant expansion of disease cases occurred in the Americas, leading to millions of suspected infections. There have also been reports of local transmission in Europe, particularly in France and Italy. Several factors, including globalisation, urbanisation, climate change, and the adaptability of mosquito vectors, have greatly contributed to the geographic spread and persistence of this disease [4].

Structure and virology of CHIKV

**Figure 2:**
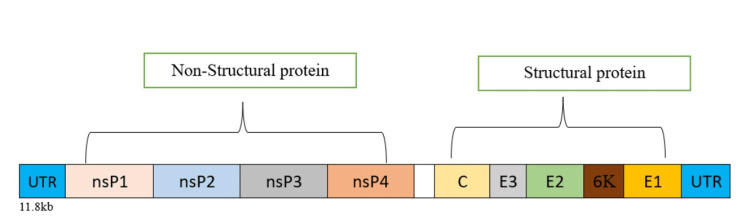
Genomic organization of chikungunya virus. URT: untranslated regions, nsP: nonstructural protein, C: capsid, E: envelope glycoprotein Source: Figure created by the authors using Microsoft Word (Microsoft Corp., Redmond, WA, USA).

CHIKV is an enveloped, positive-sense single-stranded RNA virus belonging to the genus *Alphavirus* in the *Togaviridae* family. It measures approximately 60 to 70 nm in diameter and has a genome that is about 11.8 kb. The genome encodes nine major proteins: four non-structural proteins (nsP1-nsP4) that are crucial for viral replication, and five structural proteins, which include the Capsid (C), E1, E2, E3 and 6K/TF. These structural proteins are involved in the assembly of the virion and the entry of the virus into host cells. The nucleocapsid of CHIKV is surrounded by a lipid envelope derived from the host, which contains E1 and E2 glycoprotein spikes. The E2 protein mediates receptor binding, while E1 facilitates membrane fusion during the infection process. CHIKV has three genotypes, including West African, Asian, and East/Central/South African genotype (ECSA). Indian Ocean lineage is a sublineage of the ECSA genotype. Notably, the Indian Ocean lineage demonstrates enhanced transmission as a result of the A226V mutation [4,8]. The genomic organisation of CHIKV is illustrated in Figure 2 [8].

Epidemiology of chikungunya in India

As summarised in Table 1, chikungunya was first reported in Kolkata, India, in 1963, followed by intermittent outbreaks throughout the 1960s and 1970s. A prolonged silent period occurred between 1980 and 2005; however, a major resurgence began in 2005-2006, affecting over a million people. Since then, chikungunya has become endemic, with periodic outbreaks, particularly during the monsoon and post-monsoon seasons. This resurgence is driven by the widespread presence of the *A. aegypti *and *A. albopictus* mosquitoes.

**Table 1:** Major outbreaks with year, cases, and states. CHIKV: chikungunya virus, ECSA: East/Central/South African genotype, COVID-19: coronavirus disease of 2019

Year	Location(s)	Genotype/Strain	Cases Reported	Epidemiology
1963	Kolkata	Asian genotype	Sporadic	First reported CHIKV outbreak in India [9].
1964-1973	Tamil Nadu, Andhra Pradesh	Asian genotype	Thousands	Multiple outbreaks in southern India [9].
1980-2005	Nationwide	-	Negligible	No major outbreaks reported [10].
2005-2010	Andhra Pradesh, Tamil Nadu, Karnataka, Maharashtra, Gujarat	ECSA genotype (A226V mutation)	A million cases are suspected	Re-emergence after 32 years [11].
2010-2012	Maharashtra	-	Recurring outbreaks	Sporadic and seasonal outbreaks post-epidemic years [12].
2017-2019	Tamil Nadu, Gujarat, Odisha	ECSA	Thousands	Localised outbreaks, increased surveillance and molecular typing [3].
2019-2021	Maharashtra, Delhi, Karnataka	ECSA	India-wide	Delhi reported over 7,000 cases; CHIKV was found co-circulating with dengue [13].
2020-2021	Underreported (COVID-19)	ECSA	Limited data	Surveillance was disrupted due to the pandemic [14].
2022-2024	Maharashtra	ECSA	Several thousand	Re-emergence post-COVID-19, urban transmission resurgence [14].

Impact of climate change and environmental factors on chikungunya in India

Climate change and environmental factors impact chikungunya transmission in India by favouring *A. aegypti *and *A. albopictus *mosquitoes. Rising temperatures, irregular rainfall, and extreme weather events lead to stagnant water accumulation in urban areas, boosting mosquito breeding. Moreover, warmer temperatures reduce the extrinsic incubation period, increasing transmission efficiency, with optimal conditions around 29°C [15]. Climate-driven ecological changes have led to the expansion of *Aedes* vectors linked to chikungunya transmission into areas that were previously unsuitable. *A. albopictus* is found in cooler and higher-altitude regions, including parts of the sub-Himalayas, while *A. aegypti *is spreading into areas that were previously unsuitable for its survival. Predictive models based on various climate scenarios indicate that this expansion may continue, potentially increasing the risk of future outbreaks. Additionally, seasonal monsoon patterns play a significant role in transmission, with peaks occurring from June to November due to heightened humidity and water accumulation [16].

Chikungunya remains endemic in India, particularly in Tamil Nadu, Karnataka, Kerala, Andhra Pradesh, Maharashtra, Gujarat, Uttar Pradesh, and Bihar, with consistently high case numbers. Factors such as high population density, inadequate drainage systems, and plentiful breeding habitats contribute to ongoing transmission [17]. Surveillance data from Tamil Nadu (2014-2018) show distinct seasonal peaks during the monsoon and post-monsoon periods, highlighting the influence of climatic variables on disease spread [15]. Furthermore, rapid urbanisation, ineffective waste management, and insufficient vector control worsen transmission. These combined climatic and environmental factors emphasise the necessity for region-specific surveillance and targeted vector control strategies [18].

Vectors of chikungunya

**Figure 3:**
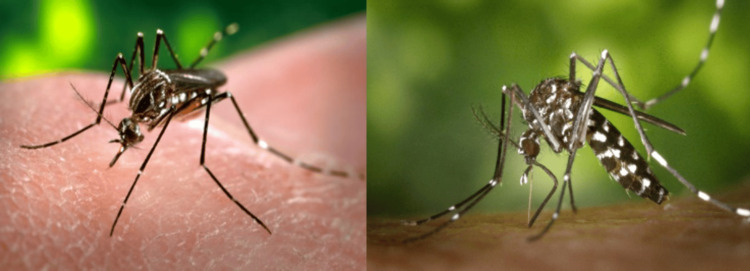
Images of Aedes aegypti (left) and Aedes albopictus (right). Source: Images obtained from Wikimedia Commons under the Creative Commons Attribution (CC BY) license [19,20].

*A. albopictus *shows flexible feeding behaviour, as it feeds on both humans and animals. This adaptability enhances its role as a bridge vector in the transmission of arboviruses. By feeding flexibly, it helps maintain viruses in animal reservoirs and increases the risk of spillover to humans, especially in peri-urban and suburban areas [17]. *A. aegypti *is highly anthropophilic, preferring to feed on humans and thriving in urban areas. Both species primarily bite during the day, peaking in the early morning and late afternoon. These differences in host preference and ecology influence chikungunya transmission dynamics in different environments [4]. In Figure 3, *A. aegypti* and *A. albopictus* are shown [19,20].

Role in rural and urban transmission of CHIKV

In India, chikungunya transmission varies between urban and rural areas due to different mosquito species. *A. aegypti *thrives in urban environments like Chennai, Bengaluru, and Mumbai, where poor waste management and artificial water storage facilitate rapid outbreaks. Chikungunya transmission in India is on the rise due to rapid urbanisation, climate variability, and the expansion of *Aedes* mosquito breeding habitats. Increasing temperatures and higher rainfall enhance mosquito survival and shorten the viral incubation period within these vectors, resulting in faster transmission cycles. Additionally, insecticide resistance and insufficient vector control measures contribute to recurring outbreaks in both urban and rural areas. *A. albopictus*, commonly found in rural and peri-urban regions, utilises natural breeding sites, allowing sustained transmission even in forested areas [21]. Rural cases often go unreported due to limited diagnostics and weak surveillance, highlighting a concealed disease burden [22]. India employs location-specific vector control strategies through integrated vector management (IVM), emphasising source reduction, environmental sanitation, and community involvement. During outbreaks, chemical methods like larvicides and space spraying are used, although resistance is growing. Biological methods, such as the use of larvivorous fish and bacterial agents, have been introduced alongside innovative tools like *Wolbachia* strategies and geographic information system (GIS)-based surveillance to enhance effectiveness. *Wolbachia*-infected mosquitoes serve as a biological control strategy, as *Aedes* mosquitoes carrying *Wolbachia* bacteria demonstrate a reduced ability to transmit chikungunya, dengue, and Zika viruses, which lowers the risk of outbreaks. Additionally, GIS and geospatial surveillance facilitate the mapping of vector breeding sites and disease hotspots. This enables targeted vector control, early warning systems, and efficient resource allocation for preventing outbreaks [1,16,23].

Clinical manifestations and complications

Chikungunya infection can be challenging to diagnose because its symptoms often resemble those of dengue fever in endemic areas. The disease usually occurs after an incubation period of two to seven days, presenting with a sudden onset of high fever and severe symmetrical joint pain (polyarthralgia), primarily affecting the hands, wrists, and ankles [24]. Common symptoms include myalgia, headache, fatigue, nausea, conjunctivitis, and a maculopapular rash on the trunk, limbs, or face. While the acute phase typically resolves in one to two weeks, many patients may develop chronic arthralgia or inflammatory arthritis similar to rheumatoid arthritis, lasting for months or years and affecting their quality of life [25]. Severe complications include neurological issues like meningoencephalitis and Guillain-Barré syndrome, along with cardiac, hepatic, and ocular involvement. These are more common in neonates, the elderly, and immunocompromised patients.

Severe chikungunya cases can involve the heart, liver, and eyes, including myocarditis, hepatitis, and retinitis, adding to the overall morbidity of the disease [26]. Disability-adjusted life years (DALY) measure the impacts of early mortality and illness, aiding in the comparison of disease burden and guiding resource allocation. Accurate data on disease prevalence and trends is crucial for policy decisions. While the Global Burden of Disease study provides DALY estimates for diseases like dengue, chikungunya lacks sufficient epidemiological data. This underscores the need for ongoing monitoring and clinical attention in endemic areas [27]. Research indicates that chikungunya is more than an acute disease, potentially leading to long-term health issues in many patients. Further research is needed to understand the causes of chronic progression and to develop targeted therapies [25].

Burden of the CHIKV on India

In India, chikungunya has a significant long-term impact beyond its initial phase. While the acute illness is typically self-limiting, about 30-60% of individuals experience persistent joint pain lasting months to years. Reports from outbreaks in Kerala and Maharashtra indicate that nearly half of patients suffer from prolonged arthralgia, particularly in the wrists, knees, and small joints, often with morning stiffness and functional limitations. These chronic symptoms resemble rheumatoid arthritis and are associated with immune-mediated inflammation and potential viral persistence [28,29]. The socio-economic impact is significant, with recurrent outbreaks causing reduced productivity, increased healthcare costs, and pressure on public health systems, especially in urban areas. Despite being preventable, gaps in vector control, surveillance, and public awareness contribute to ongoing transmission. This suggests that many cases could be avoided with improved public health interventions and timely management strategies [29,30].

Challenges of chikungunya control in India

In India, controlling chikungunya is challenging due to its co-circulation with dengue fever and Zika virus, which causes overlapping clinical symptoms and diagnostic difficulties [31]. Molecular methods such as RT-PCR offer high sensitivity during the acute phase (the first five to seven days), while serological tests like enzyme-linked immunosorbent assay (ELISA) are used later. However, these tests can produce false positives due to cross-reactivity with other arboviruses [32]. Rapid diagnostic tests and point-of-care tools have significantly improved early detection of diseases. However, their sensitivity and accessibility remain limited in low-resource settings. Co-infections can complicate both diagnosis and management. Recent advancements, such as reverse transcriptase loop-mediated isothermal amplification (RT-LAMP), antigen assays, and genomic sequencing, have enhanced our ability to detect infections and understand viral diversity. Additionally, novel techniques like quantum-dot-based assays show promise for improved sensitivity. Despite these advancements, inadequate molecular diagnostic infrastructure and resource constraints still hinder effective surveillance and outbreak control [31].

Vector control and patient management

Effective control of chikungunya relies on IVM and supportive patient care. Source reduction remains the primary strategy, involving the elimination of stagnant water in containers such as tyres, tanks, and drains, supported by community-based sanitation efforts. Biological control methods, including larvivorous fish like *Gambusia affinis* and *Poecilia reticulata*, and bacterial larvicides such as *Bacillus thuringiensis israelensis* (Bti), offer eco-friendly alternatives [30]. However, challenges like insecticide resistance, poor infrastructure, and limited public awareness hinder effectiveness. Personal protective measures, repellents, protective clothing, and screened spaces further reduce mosquito exposure. Patient management is mainly symptomatic, as no specific antiviral therapy exists. Antipyretics (e.g. paracetamol) and nonsteroidal anti-inflammatory drugs (NSAIDs) are used after excluding dengue fever [33]. Rest and hydration are essential during the acute phase. Chronic arthralgia may persist for months, requiring long-term monitoring, supportive care, and rehabilitation to improve quality of life [34].

Economic burden and research gaps in India

Chikungunya has become a significant public health issue in India, leading to considerable clinical and economic burdens. However, comprehensive and up-to-date data on its economic impact are lacking. Affected households face heavy financial strains from direct costs like hospital stays and medications, as well as indirect costs such as missed work and long-term disability. Studies indicate that chikungunya is underrepresented in economic evaluations compared to dengue or malaria, especially affecting lower-income groups. There is also a notable lack of localised cost-of-illness studies for chikungunya in India [35]. Their systematic review on the economic impact of chikungunya highlights that most research focuses on Latin America, with limited data from South Asia, particularly India. While some state-level figures exist, national-level estimates considering home and health system costs are scarce. This gap is crucial for informing budgeting for vector management and public health strategies during epidemics [36,37].

Socio-economic impact and health system strain

Chikungunya epidemics cause significant socioeconomic disruptions in addition to acute public health issues, particularly in low- and middle-income countries. The combination of high morbidity, long recovery times, and recurring outbreaks places a heavy burden on individuals, families, and national health systems. The direct costs associated with chikungunya infection can be substantial, including healthcare expenses for hospitalisation, outpatient consultations, diagnostic tests (such as PCR and serology), and symptomatic treatments like analgesics and anti-inflammatory medications. These costs are often covered by both public and private healthcare facilities, whether in endemic areas or during outbreaks. Moreover, during major outbreaks, public health officials may have to allocate emergency funds for patient care and vector control, diverting resources from other essential services [38]. Chikungunya outbreaks result in high costs for patients, families, and healthcare systems. Persistent symptoms such as joint pain and fatigue reduce productivity, lead to absenteeism, and strain households, particularly among day labourers and non-regular workers. Sectors such as agriculture and construction may also be impacted. Health systems face challenges, including staff shortages and hospital congestion, especially when CHIKV coincides with dengue and Zika. Chronic symptoms can negatively affect mental health and quality of life, with some patients experiencing long-lasting effects [38,39].

## Conclusions

In India, chikungunya is a major public health concern due to its high morbidity, frequent outbreaks, and long-term health effects like chronic arthralgia. There are still significant gaps in long-term patient treatment, surveillance, and diagnostic capability despite improvements in clinical recognition and vector control techniques. Numerous factors, such as expanding mosquito habitats, increased human mobility, and rapid urbanisation, are contributing to the virus's resurgence. To reduce the impact of chikungunya in the future, it is essential to invest in vaccine development, improve genetic surveillance, and strengthen IVM. Effective strategies to reduce disease transmission and enhance public health resilience against this often-overlooked arboviral threat require a coordinated effort across multiple sectors.
